# Structure-based Inhibitor Design for the Intrinsically Disordered Protein c-Myc

**DOI:** 10.1038/srep22298

**Published:** 2016-03-02

**Authors:** Chen Yu, Xiaogang Niu, Fan Jin, Zhirong Liu, Changwen Jin, Luhua Lai

**Affiliations:** 1 BNLMS, State Key Laboratory for Structural Chemistry of Unstable and Stable Species, College of Chemistry and Molecular Engineering, Peking University, Beijing 100871, China; 2 Beijing Nuclear Magnetic Resonance Center, Peking University, Beijing 100871, China; 3 College of Chemistry and Molecular Engineering, Peking University, Beijing 100871, China; 4 Center for Quantitative Biology, Peking University, Beijing 100871, China; 5 Peking-Tsinghua Center for Life Sciences, Peking University, Beijing 100871, China

## Abstract

Intrinsically disordered proteins (IDPs) are associated with various diseases and have been proposed as promising drug targets. However, conventional structure-based approaches cannot be applied directly to IDPs, due to their lack of ordered structures. Here, we describe a novel computational approach to virtually screen for compounds that can simultaneously bind to different IDP conformations. The test system used c-Myc, an oncoprotein containing a disordered basic helix-loop-helix-leucine zipper (bHLH-LZ) domain that adopts a helical conformation upon binding to Myc-associated factor X (Max). For the virtual screen, we used three binding pockets in representative conformations of c-Myc_370–409_, which is part of the disordered bHLH-LZ domain. Seven compounds were found to directly bind c-Myc_370–409_ *in vitro*, and four inhibited the growth of the c-Myc-overexpressing cells by affecting cell cycle progression. Our approach of IDP conformation sampling, binding site identification, and virtual screening for compounds that can bind to multiple conformations provides a useful strategy for structure-based drug discovery targeting IDPs.

Discovered in the 1990s, intrinsically disordered proteins (IDPs) abolished the dogma that a folded three-dimensional structure is necessary for the biological function of a protein^1^,^2^,^3^,^4^,^5^,^6^. These proteins have minimal structures and exist as dynamic ensembles under physiological conditions. IDPs are highly abundant in numerous genomes^2^,^3^,^4^,^5^. Computational analyses have predicted the level of IDPs as approximately 40% of eukaryotic, 25% of viral, 10% of bacterial, and 10% of archaeal proteins^4^. Despite being highly flexible and lacking stable secondary and tertiary structures, IDPs have crucial biological functions in protein-DNA/RNA recognition, transcriptional activation, translation, cell signalling, cell cycle progression, and enzyme allosteric regulation^1^,^6^,^7^,^8^. Furthermore, 52% to 67% of human proteins associated with signalling, cancer, cardiovascular disease, neurodegenerative disease, and diabetes have been predicted to be disordered, making IDPs potential drug targets^9^. However, the lack of ordered structures in IDPs is an inherent challenge in using them as druggable targets by conventional structure-based rational drug design approaches.

Three strategies can be used to disrupt the biological functions of IDPs^10^. First, molecules that bind to the ordered domains of IDPs can be used to modulate their functions. Second, molecules that bind to the ordered partner protein of an IDP can be used to block its binding (e.g., p53-Mdm2 inhibitors). In both cases, drug design can target the ordered protein using conventional drug design methods. The third strategy involves directly targeting the functional disordered region of IDPs. Functional misfolding can spontaneously develop in some IDPs, thus preventing them from interacting with non-native partners. Therefore, stabilizing the functionally misfolded structures of IDPs is an effective alternative method to directly targeting IDPs and can be implemented with a rational method^11^. Similarly, targeting aggregation structures or stabilizing the non-amyloidogenic oligomeric or monomeric species of IDPs can also be accomplished^12^,^13^,^14^. The most difficult approach is to directly target the conformation ensembles of IDPs, which is attractive because molecules binding to the dynamic IDPs can directly prevent their biological interactions. However, this is very challenging as conventional “rational drug design” methods cannot be used without a well-defined target structure^3^,^10^.

Although IDPs have been proposed as potential targets for drug discovery, few rational drug design methods directly targeting IDP disordered states have been reported thus far. Examples that used high-throughput experimental screening to target IDPs are also limited. Using a yeast two-hybrid system to screen a 10,000-member diversity library, *Yin, et al.* identified seven Myc-Max-specific low-molecular-weight inhibitors, which inhibit cell cycle progression and fibroblast growth^15^. Further circular dichroism and nuclear magnetic resonance experiments identified three different binding sites (c-Myc_402–409_ for 10058-F4, c-Myc_366–375_ for 10074-G5, and c-Myc_375–485_ for 10074-A4) located within the disordered bHLH-LZ domain of c-Myc^16^,^17^. These small molecules can bind c-Myc and stabilize the intrinsically disordered monomer over the highly ordered c-Myc-Max heterodimer^16^,^17^,^18^. This example demonstrates that small molecules can be used to modulate IDP function and directly target IDPs even though their binding sites were identified in a later study. A generally applicable rational drug design strategy for IDPs will open a new door to make large numbers of IDPs druggable^19^. One option is to target some native-like models of IDPs, predicted either by studying protein folding mechanisms *in silico* or through *ab initio* modelling based on the lowest free energy state identified^20^. Alternatively, some cases of aggregating IDPs have shown that targeting one particular conformation obtained from clusters of molecular dynamics simulations can be effective^13^,^14^. Because the energy landscape of IDPs is much flatter than the funnel-shaped landscape of well-folded globular proteins^21^, one can expect that small molecules targeting IDPs may bind to various conformations of IDPs. However, these ideas still lack sufficient experimental evidence.

The intrinsically disordered protein c-Myc is a transcription factor that regulates the expression of various genes involved in cell proliferation, differentiation, metabolism, adhesion, apoptosis, maintenance of cell size, genomic integrity, and angiogenesis^9^,^22^,^23^. A member of the bHLH-LZ family, heterodimerization of c-Myc with its partner Max, which is also a bHLH-LZ protein, is essential for DNA binding and transcriptional activation^14^,^15^,^16^,^22^. Because c-Myc is overexpressed in many human cancers, such as breast cancer, colon cancer, cervical cancer, small-cell lung carcinomas, osteosarcomas, glioblastomas, melanoma, and myeloid leukaemias^22^,^23^,^24^, it is an attractive anti-cancer target. However, disrupting c-Myc-Max dimerization is difficult because both proteins are IDPs and the protein-protein interface is flat and lacks recognizable motifs^24^,^25^,^26^. Interestingly, single amino acid substitutions within the bHLH-ZIP dimerization domain of c-Myc abolished its interaction with Max and abrogated its transcriptional activation function and biological properties^27^. These observations make targeting c-Myc even more appealing.

Similar to binding sites in ordered proteins, analysis on cavities in IDPs also found druggable properties^28^. In a previous study, we used a computational approach to study the binding characteristics of one reported c-Myc binding compound, 10074-A4. We found that this compound associates with c-Myc_370–409_ and behaves like a “ligand cloud” around a “protein cloud”, with distinct features from that of a non-binding ligand^29^. We hypothesized that representative conformations of IDP and multiple binding sites within them can be used to virtually screen for potential binding molecules. This hypothesis was tested here in the c-Myc system to discover molecules that can specifically bind to the disordered bHLH-LZ domain of c-Myc. Two typical conformations of c-Myc_370–409_ and three predicted binding sites within them were used for the virtual screen. We discovered four active compounds that bind c-Myc_370–409_ and block its function in the cell. To our knowledge, this is the first successful example using a structure-based approach to discover molecules that directly target the c-Myc conformation ensemble.

## Results

### Virtual screen

The Apo and Holo conformations of c-Myc_370–409_ from our previous computational work were used as two typical conformations^29^. Potential binding site analysis using the CAVITY program^30^ identified two pockets in the Apo conformation (here named cavities Apo1 and Apo2), and one pocket in the Holo conformation (cavity Holo1). These three predicted binding sites were used to screen for potential binding compounds. Compounds in the SPECS^31^ and DCSD (a small in-house library of the Peking University School of Pharmaceutical Sciences) libraries were docked into the three potential binding sites using Glide^32^,^33^ SP mode. In addition, both the S and R forms of 10074-A4 were used for a compound similarity search using Phase^34^. In total, 250 compounds from the virtual screen and 23 analogues from the similarity search were purchased from the SPECS and DCSD libraries for activity testing.

### *In vitro* binding test

**Figure 1:**
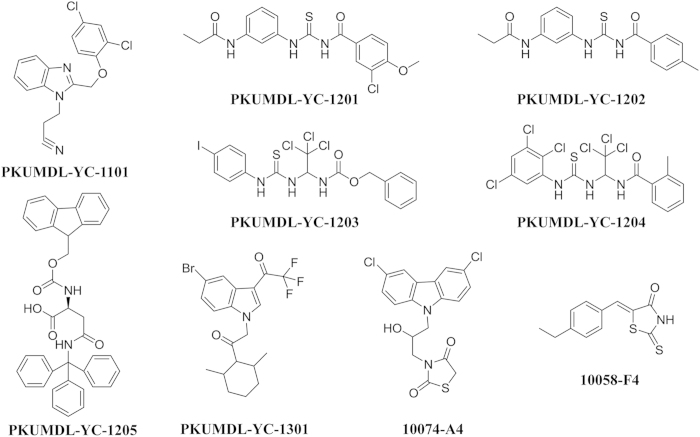
Chemical structures of the active compounds.

**Figure 2:**
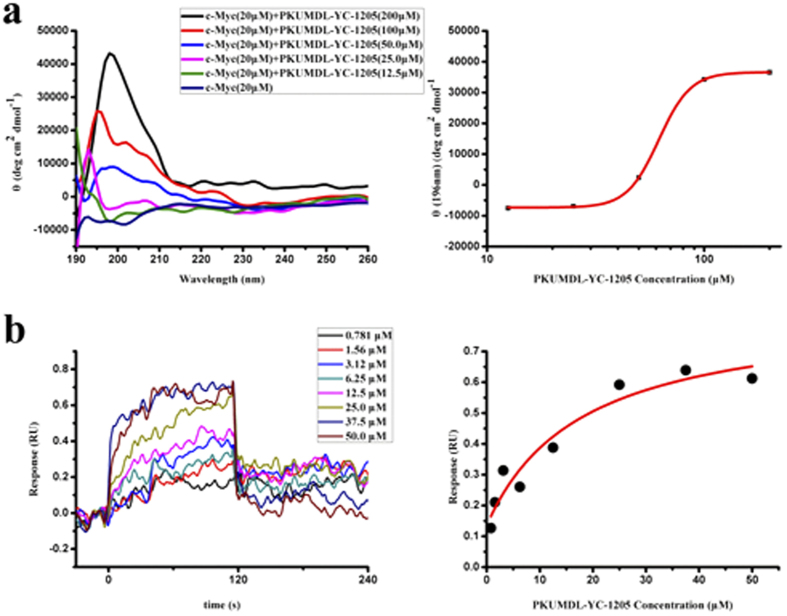
Activities of PKUMDL-YC-1205 in cell-free assays. (**a**) CD spectra of c-Myc_370–409_ with indicated concentrations of PKUMDL-YC-1205 (left) and dose-response curve at 196 nm (right). The apparent K_d_ of PKUMDL-YC-1205 was 61.8 ± 0.7 μM, as predicted by the Hill equation. (**b**) SPR direct binding curves of the indicated concentrations of PKUMDL-YC-1205 (left). The K_d_ value was 18 ± 12 μM based on affinity fitting of the dose-response curve (right). Data represent the mean ± standard deviation of three independent experiments.

The 273 selected compounds were first tested using a published procedure^17^ for their abilities to cause a CD spectra change of c-Myc_370–409_. Seven compounds induced significant local changes at different wavelengths in the CD spectra in a concentration-dependent manner (Figs 1 and 2a and Supplementary Fig. S1). The activity of these compounds was quantified as apparent K_d_ using the Hill equation. All seven compounds exhibited good activity (Table 1 and Supplementary Fig. S1) with apparent K_d_ values of 94 ± 21 μM, 70 ± 11 μM, 90 ± 15 μM, and 61.8 ± 0.7 μM for PKUMDL-YC-1101, PKUMDL-YC-1201, PKUMDL-YC-1204, and PKUMDL-YC-1205, respectively. As a control, we also measured the apparent K_d_ value of 10074-A4, which was 128 ± 46 μM (Supplementary Fig. S1). Six of the seven compounds were from the SPECS library whereas PKUMDL-YC-1205 was from the DCSD library. PKUMDL-YC-1101 was discovered using cavity Apo1, whereas PKUMDL-YC-1201, PKUMDL-YC-1202, PKUMDL-YC-1203, PKUMDL-YC-1204, and PKUMDL-YC-1205 were found using cavity Holo1. PKUMDL-YC-1301 is an analogue of 10074-A4 (Table 1, Supporting Information).

The binding strength of these seven compounds was measured further using surface plasmon resonance (SPR). After a c-terminal biotinylated c-Myc_370–412_ peptide was immobilized onto a SA chip, serial concentrations of compounds were injected. The binding signals were continuously recorded in response units (RU) and presented graphically as a function of time. As shown in Fig. 2b and Supplementary Fig. S2, all seven compounds bound biotinylated c-Myc_370–412_ in a concentration-dependent manner. PKUMDL-YC-1101, PKUMDL-YC-1201, PKUMDL-YC-1203, PKUMDL-YC-1204, and PKUMDL-YC-1205 showed better binding affinity (with K_d_ of 0.28 ± 0.14 μM, 17.2 ± 7.2 μM, 32 ± 18 μM, 0.55 ± 0.14 μM, and 18 ± 12 μM, respectively) than 10074-A4 (K_d_ 36.3 ± 9.0 μM, Table 1).

### Cell-based assays

**Figure 3:**
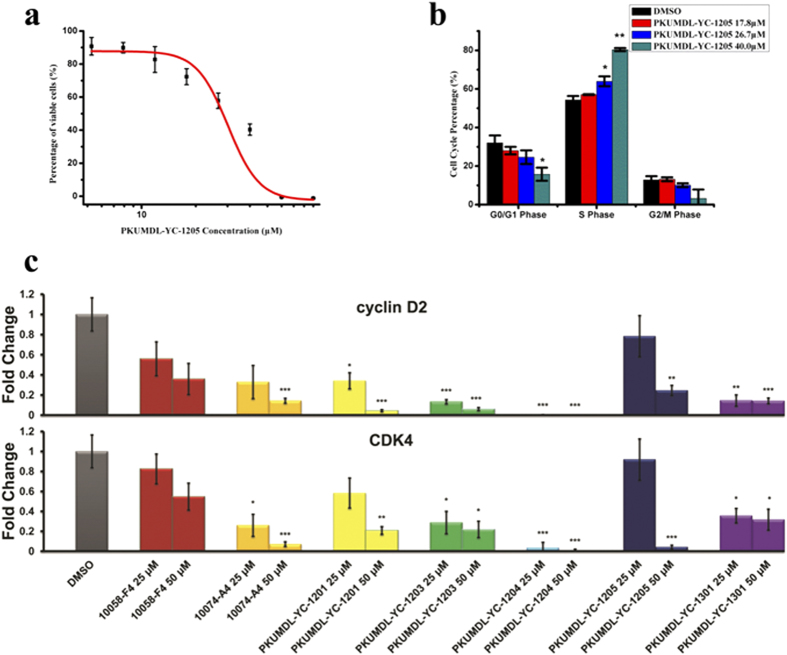
Activities of PKUMDL-YC-1205 in cell-based assays. (**a**) Growth inhibition of HL-60 cells by PKUMDL-YC-1205 was assessed by the MTT assay after exposure to indicated concentrations of PKUMDL-YC-1205 for 72 hours. The EC_50_ was 40.0 ± 1.9 μM. (**b**) Percentage of HL-60 cells in different phases of the cell cycle after treatment with 17.8 μM, 26.7 μM, and 40.0 μM PKUMDL-YC-1205 for 24 hours. Data represent the mean ± standard deviation of three independent experiments. (**c**) c-Myc mediated transcriptional activity in HL-60 cells was blocked. Data represent the mean ± standard error of three independent experiments. *p < 0.05, **p < 0.01, ***p < 0.005.

Overexpression of the c-Myc gene in the human promyelocytic leukaemia cell line HL-60 is pivotal in carcinogenesis. The MTT assay has been used to test the inhibitory activity of 10058-F4 and other compounds on HL-60 cell growth^24^,^35^,^36^,^37^,^38^,^39^,^40^,^41^. We also used this assay to examine whether these seven compounds abrogate cell proliferation. Our results demonstrate that HL-60 cells were sensitive to four of the seven compounds in a dose-dependent manner (Fig. 3a, Supplementary Fig. S3, and Table 1). PKUMDL-YC-1203, PKUMDL-YC-1204, and PKUMDL-YC-1205 showed EC_50_ values in the micromolar range (6.9 ± 1.1 μM, 8.80 ± 0.26 μM, and 40.0 ± 1.9 μM, respectively), which are comparable to or better than 10074-A4 (15.1 ± 2.3 μM) and 10058-F4 (40.3 ± 2.7 μM, Table 1).

We further examined whether the reduced cell numbers observed in the MTT assay were caused by cytotoxicity or by an effect on cell cycle progression. After 24-hour treatment with different concentrations of compounds below the EC_50_ value (as determined at 72 hours), HL-60 cells were harvested for cell cycle analysis by flow cytometry. PKUMDL-YC-1201, PKUMDL-YC-1203, PKUMDL-YC-1204, PKUMDL-YC-1205, PKUMDL-YC-1301, 10074-A4, and 10058-F4 arrested the cell cycle at the S-phase in a dose-dependent manner (Fig. 3b, Supplementary Fig. S4, and Table 1). These compounds led to a significantly higher percentage of S-phase cells and lower frequency of G_2_/M-phase cells at low concentrations.

In order to confirm that the cell cycle progression arrest is caused by the inhibition of c-Myc, changes in the mRNA level of the c-Myc target genes, *CCND2* and *CDK4* (encoding proteins cyclin D2 and CDK4), were evaluated by quantitative real-time PCR. The expression of c-Myc dependent genes was decreased in cells treated with PKUMDL-YC-1201, PKUMDL-YC-1203, PKUMDL-YC-1204, PKUMDL-YC-1205, PKUMDL-YC-1301, 10074-A4, and 10058-F4 compared with that in controls (Fig. 3c and Table 1).

### PKUMDL-YC-1205 blocks the interaction between c-Myc_370–409_ and Max

**Figure 4:**
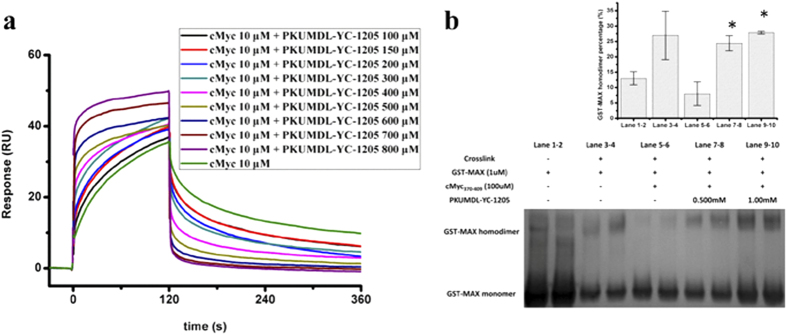
PKUMDL-YC-1205 blocks the interaction between c-Myc_370–409_ and Max. (**a**) PKUMDL-YC-1205 abolished cMyc_370–409_ binding to Max in the SPR competitive assay at the indicated concentrations. (**b**) PKUMDL-YC-1205 disrupted the Max-Max/c-Myc-Max dimerization equilibrium. Chemical cross-linking and anti-Max western blotting results are shown (left). Blackness integrals of the GST-Max homodimer percentage are shown as a histogram (right). Data represent the mean ± standard deviation of two independent experiments. *p < 0.05.

Because PKUMDL-YC-1203 and PKUMDL-YC-1204 are less soluble in water, we performed a detailed binding analysis using PKUMDL-YC-1205. To investigate the influence of PKUMDL-YC-1205 on the heterodimerization of c-Myc_370–409_ with its partner Max, an SPR competitive binding assay was used. After immobilization of GST-Max onto a CM5 chip by amine-coupling, serial concentrations of PKUMDL-YC-1205 pre-incubated with c-Myc_370–409_ were injected automatically. As shown in Fig. 4a, PKUMDL-YC-1205 greatly reduced c-Myc_370–409_ binding to GST-Max in the c-Myc_370–409_ dissociation curves. The observed increase in steady state response as the PKUMDL-YC-1205 concentration in the injection mixture increased can be attributed to non-specific binding of PKUMDL-YC-1205 to GST-Max (Supplementary Fig. S5). As expected, c-Myc_370–409_ showed no binding to GST in the SPR assay.

The influence of PKUMDL-YC-1205 on the Max-Max/c-Myc-Max dimerization equilibrium was also tested. Chemical cross-linking experiments were conducted with GST-Max and GST as control in the presence and absence of c-Myc_370–409_ and PKUMDL-YC-1205. Figure 4b shows that PKUMDL-YC-1205 decreased the c-Myc-Max heterodimer ratio, consequently leading to an increased Max homodimer ratio. PKUMDL-YC-1205 produced no effect on the GST dimerization equilibrium experiments (Supplementary Fig. S6).

### The binding mode of PKUMDL-YC-1205 with c-Myc_370–409_

**Figure 5:**
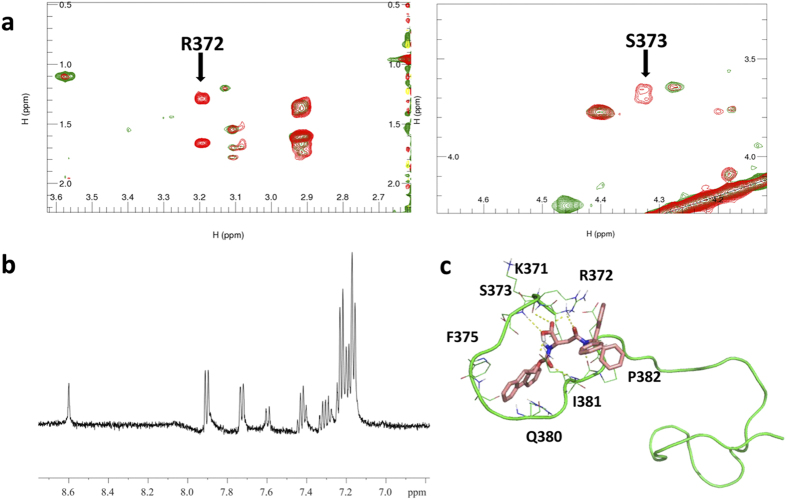
NMR study of PKUMDL-YC-1205 binding with c-Myc_370–409_. (**a**) Partially enlarged details of overlapped ^1^H-^1^H TOCSY spectrum of c-Myc_370–409_ with (green) and without (red) PKUMDL-YC-1205. (**b**) STD NMR spectrum of PKUMDL-YC-1205 with c-Myc_370–409_ (molar ratio 50:1). (**c**) One binding model of c-Myc_370–409_ with PKUMDL-YC-1205. c-Myc_370–409_ is represented in green and PKUMDL-YC-1205 is represented in pink.

We used nuclear magnetic resonance (NMR) spectroscopy to characterize the binding features of PKUMDL-YC-1205 to c-Myc_370–409_. Comparison of the TOCSY spectra showed that the cross peaks of Arg372 Hβ-Hγ-Hδ and Ser373 Hα-Hβ disappeared after the addition of PKUMDL-YC-1205 (Fig. 5a and Supplementary Fig. S7), suggesting that PKUMDL-YC-1205 interacts with c-Myc_370–409_ near Arg372 and Ser373.

To confirm the interactions between PKUMDL-YC-1205 and c-Myc_370–409_, a Saturation-Transfer Difference (STD) NMR experiment was performed. In the STD experiment, the ^1^H resonances of the large molecule (c-Myc_370–409_) were selectively irradiated and subsequent magnetization was transferred to the ^1^H of the small molecule (PKUMDL-YC-1205). The differences in the spectrum derived after subtracting the reference spectrum in which the large molecule ^1^H are not irradiated, and hence no magnetization transfer occurs, identifies which of the ^1^H of the small molecule are in closest contact with the large molecule in the bound state^42^,^43^. We prepared one sample of 2 mM PKUMDL-YC-1205 mixed with 0.04 mM c-Myc_370–409_ in DMSO-d_6_ and a reference sample only containing 2 mM PKUMDL-YC-1205. Selective irradiation was placed at approximately 0.84 ppm, which is the methyl ^1^H region of c-Myc_370–409_. Figure 5b depicts the STD NMR spectrum of PKUMDL-YC-1205 with c-Myc_370–409_. It is clear that the selective irradiation from c-Myc_370–409_ made ^1^H resonances of PKUMDL-YC-1205 appear in a different spectrum, indicating that c-Myc_370–409_ interacts with PKUMDL-YC-1205 and allows for saturation to be transferred from c-Myc_370–409_ to PKUMDL-YC-1205 in the bound state. The signals are derived from hydrogens from the aromatic rings and secondary amines of PKUMDL-YC-1205. Thus, the STD NMR experimental results support that PKUMDL-YC-1205 binds with c-Myc_370–409_ (Fig. 5c).

**Figure 6:**
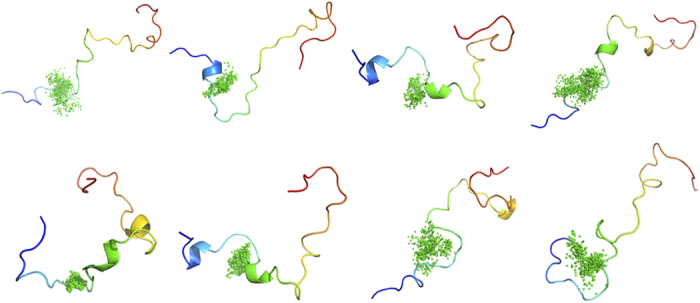
PKUMDL-YC-1205 binds to multiple conformations of c-Myc_370–409_ in molecular dynamics simulations. Conformations from the five simulations of c-Myc_370–409_ with PKUMDL-YC-1205 were clustered. The c-Myc_370–409_ structures are depicted in a rainbow (from blue at the N-terminal to red at the C-terminal) and PKUMDL-YC-1205 structures are depicted at the centres of mass as green dots.

Furthermore, we investigated the interactions between PKUMDL-YC-1205 or 10074-A4 (the S form) and c-Myc_370–409_ using molecular dynamics simulations. The initial complex structures were built by molecular docking and then five independent 100-nanosecond simulations were performed. Even though the compounds were residing in their binding sites in the initial structures, they could hover along the c-Myc_370–409_ structure during the simulation course (Fig. 6). The interaction distances between the compounds and residues in c-Myc_370–409_ were calculated and used to characterize the binding site (within 5 Å). PKUMDL-YC-1205 and 10074-A4 showed a strong tendency to bind to the N-terminal residues 370–387 and 375–385 in c-Myc_370–409_ (Supplementary Fig. S8), respectively.

## Discussion

By using a structure-based virtual screen approach, we successfully discovered seven molecules that bind directly to the c-Myc disordered bHLH-LZ domain and inhibit c-Myc-Max dimerization. Four compounds exhibited micromolar binding affinity and inhibited cancer cell growth. None of the compounds appeared to match the list of PAINS compounds, therefore, they may not be promiscuous^44^. No biological activities have been reported for PKUMDL-YC-1101, PKUMDL-YC-1201, PKUMDL-YC-1202, PKUMDL-YC-1203, PKUMDL-YC-1204, and PKUMDL-YC-1301. PKUMDL-YC-1205, an amino acid derivative, was reported to weakly inhibit botulinum neurotoxin A protease in a cellular model (IC_50_ = 82 ± 7 μM), as well as block γ-secretase (IC_50_ = 17.5 μM)^45^,^46^. None of these compounds were reported previously to inhibit c-Myc activity. To the best of our knowledge, this is the first successful example that used a structure-based design approach to discover compounds that directly bind to the c-Myc conformation ensemble and inhibit its biological function.

Only a few compounds that inhibit c-Myc-Max dimerization have been reported in the last decade. In 2002, Berg *et al.* found several peptide mimetic compounds as the first small molecule inhibitors of c-Myc-Max dimerization using high-throughput experimental screening, and the best compound, IIA6B17, showed IC_50_ values of 125 ± 25 μM by ELISA and 50 ± 25 μM by EMSA^31^. In 2003, Yin *et al.* discovered seven small molecules that can disrupt the c-Myc-Max interaction using a yeast two-hybrid approach, including 10058-F4, 10074-A4, and 10074-G5^15^. Synthesized derivatives of 10058-F4 showed improved IC_50_ of HL-60 cell growth inhibition from 49 μM (10058-F4) to 4.6 μM (#474)^36^. Meanwhile, the prodrug of JY-3-094 (an analogue of 10074-G5), which penetrates cells poorly, improved the IC_50_ of HL-60 cell growth from 30 μM (10074-G5) to 7.2 μM (3JC-91-2)^40^,^47^. Similarly, after screening a pyrazolo[1,5-α]pyrimidine library based on the structures of Mycro1 and Mycro2, which were identified from a fluorescence polarization high-throughput screen, Mycro3 was found with enhanced activity (IC_50_ for TGR-1 (*Myc*+/+) cells was 0.25 μM)^48^,^49^,^50^. Recently, through a fluorescence polarization screen for the Myc-Max interaction, KJ-Pyr-9, from a Kröhnke pyridine library, was shown to inhibit the proliferation of NCI-H460, MDA-MB-231, and SUM-159PT cells with IC_50_ values from 5 to 10 μM^51^.

Among all these inhibitors, only 10058-F4, 10074-A4, 10074-G5, and KJ-Pyr-9 have been shown to bind directly to c-Myc by CD, NMR, or backscattering interferometry^16^,^17^,^51^. The binding sites of 10058-F4, 10074-A4, and 10074-G5 were shown to be located within the disordered bHLH-LZ domain of c-Myc. The K_d_ values of 10058-F4, #474, and 10074-G5 binding to the bHLH-LZ domain of c-Myc based on SPR experiments were reported as 39.7 ± 8.1 μM, 16.6 ± 1.4 μM, and 31.7 ± 24.9 μM, respectively^52^.

Compared to the high-throughput screening approaches used previously, our strategy directly targets the disordered bHLH-LZ domain of c-Myc, which is more efficient. Experimental testing of the 273 compounds predicted by the computational screen identified seven active ones.

Another question that arises from directly targeting IDPs with small molecules is whether the binding is truly specific. Our computational studies demonstrated binding sequence specificity for both PKUMDL-YC-1205 and 10074-A4. Additionally, the flexibility of the disordered region makes it possible for an IDP to bind a diversity of conformations of one compound with similar affinities. This promiscuous “ligand clouds around protein clouds” binding mode^29^ may have advantages in binding kinetics over the “lock and key” and induced-fit binding modes. Moreover, PKUMDL-YC-1205 and 10074-A4 had longer binding times with c-Myc_370–409_ compared to the non-active compound AJ-292/41944612 (shown by molecular dynamics simulations, Supplementary Fig. S8). The docking scores of AJ-292/41944612 with the three pockets were −6.307, −2.766, and −3.072 to cavity Apo1, Apo2, and Holo1, respectively. Therefore, the non-active compound AJ-292/41944612 may only bind to a small fraction of conformations. In contrast, all six active compounds obtained from molecular docking, as well as 10074-A4, are “multi-conformational-affinity” compounds (i.e., compounds that bind to various groups of conformations with similar affinity). Therefore, IDPs may have a tendency to bind to “multi-conformational-affinity” compounds instead of “high-conformational-specificity” ones (i.e., compounds with high affinity to one class of conformation but very low affinity to others).

We performed a structure-activity analysis with four compounds, namely PKUMDL-YC-1201, PKUMDL-YC-1202, PKUMDL-YC-1203, and PKUMDL-YC-1204, which share a common thiourea structure. All four compounds formed hydrogen bonds with the backbone oxygen atom from Glu383 through the two hydrogen atoms of the thiourea group. They also formed a hydrogen bond with the backbone oxygen atom from Asp379 via the hydrogen from the acylamino group in the docking mode (Supplementary Fig. S9). PKUMDL-YC-1203 and PKUMDL-YC-1204 can use thiourea and acylamino groups to form additional hydrogen bonds with the backbone oxygen atom from Ile381. Furthermore, PKUMDL-YC-1201, PKUMDL-YC-1202, and PKUMDL-YC-1203 form hydrogen bonds with the side chain of Arg378. The propiono group of PKUMDL-YC-1201 is better oriented to form good hydrophobic interactions with the benzene ring of Phe375 (Supplementary Fig. S9), which makes PKUMDL-YC-1201 more active than PKUMDL-YC-1202. The distance between the oxygen from the benzyloxy group of PKUMDL-YC-1203 and the backbone oxygen atom from Asp379 is only 3.2 Å (Supplementary Fig. S9), resulting in electrostatic repulsion that makes PKUMDL-YC-1203 less active than PKUMDL-YC-1204. Further optimization of these compounds can be performed based on this analysis.

Although directly targeting IDPs and disrupting ID-based protein-protein interaction is a relatively new strategy, previous studies^16^,^17^ and our work demonstrate that “protein clouds” are indeed druggable. Disrupting the c-Myc-Max protein-protein interaction by binding to the bHLH-LZ domain of c-Myc is an important strategy in designing and screening for c-Myc-Max inhibitors. To test whether computer-aided drug design targeting an intrinsically disordered region is feasible, we performed a virtual screen for compounds that can bind to free and bound conformations of c-Myc_370–409_. Four compounds (PKUMDL-YC-1201, PKUMDL-YC-1203, PKUMDL-YC-1204, and PKUMDL-YC-1205) showed micromolar affinity in our *in vitro* binding and cell-based assays, demonstrating the feasibility of directly targeting IDPs using a structure-based drug design method.

Therefore, based on our study, we propose a general approach for directly targeting IDPs. First, comprehensive conformation sampling should be performed for the IDPs and some representative or lower energy conformations should be selected for virtual screening. Next, we suggest a chemical library screen through multi-conformational molecular docking, followed by docking score analysis and “multi-conformational-affinity” compound selection. Finally, various experimental methods can be used to validate activity.

In conclusion, we have used a general approach for structure-based discovery of IDP binding molecules to discover compounds that bind to the c-Myc disordered bHLH-LZ domain and prevent c-Myc-Max dimerization. Seven compounds were successfully discovered and four of them were active in cell-based assays. Because many IDPs are related to human diseases, this approach provides a useful tool to make them truly druggable.

## Experimental Section

### Materials

The selected compounds were from the SPECS and DCSD libraries with purity of more than 90% and for most compounds greater than 95%. The compounds from SPECS were confirmed by the supplier, using NMR, LC-MS, or both; data are available through the Web site (see Supporting Information for the order numbers). Compound PKUMDL-YC-1205 was bought from Shenzhen Biochemilogic Technology Co. Ltd. and was confirmed by the supplier using NMR, LC-MS, and elemental analysis.

### Binding site prediction

We used CAVITY, a program for binding site detection and druggability assessment, to predict potential binding sites in c-Myc_379–409_^30^. Two sites in the Apo conformation and one in the Holo conformation were identified: cavity Apo1 in c-Myc_379–409_, Apo2 in c-Myc_370–386_, and Holo1 in c-Myc_374–388_.

### Virtual screen

Molecular docking studies were performed using Glide^32^,^33^. The SP mode of Glide was used to screen the entire DCSD and SPECS libraries. After molecular docking, the top 5% of docked compounds for the different cavities were chosen for manual selection according to the following criteria: (1) formation of at least one hydrogen bond to residues in their best score-binding conformation, and (2) formation of good hydrophobic interactions with residues in their best score-binding conformation. The selected compounds can be divided into two classes: (1) “high-conformational-specificity” compounds, where the best docking score among the three cavities is less than −6.000 and the other two are greater than −4.000, and (2) “multi-conformational-affinity” compounds, where the differences of the three scores are less than 2.0 and at least one of the three docking scores is less than −5.000. 10074-A4 belongs to the second class (Table 1). Analogue screening of 10074-A4 was conducted using Phase^34^.

### Circular dichroism

For the preliminary activity test, samples of c-Myc_370–409_ (20 μM, synthesized by GL Biochem Ltd., Shanghai, China) in the absence and presence of the compounds (at 100 μM) were prepared in 10 mM potassium phosphate, 100 mM potassium chloride, pH 7.4. The compounds were added from stock solutions in ethanol. CD spectra were recorded using a 1-mm path-length quartz cuvette at 25 °C on a BioLogic MOS-450 AF/AF-CD spectropolarimeter. For compounds active at 100 μM, further tests were done using various concentrations of the compounds. For each CD experiment, the corresponding concentration of compound was used as the blank. The apparent K_d_ was calculated using the Hill equation.

### Surface plasmon resonance (SPR)

SPR experiments were performed using a Biacore T200 (GE Healthcare Biacore, Uppsala, Sweden) instrument at 25 °C. In the direct binding assay, c-terminal biotinylated c-Myc_370–412_ (synthesized from GL Biochem Ltd.) was immobilized onto an SA chip (GE Healthcare) to a level of approximately 500 response units (RU). In the competitive binding assay, GST-Max (Sino Biological Inc., Beijing, China) was immobilized onto a CM5 chip (GE Healthcare) to a level of approximately 500 RU. The experiments were performed in 1X PBS-P (0.05% or 0.5% P20, GE Healthcare) with 5% DMSO. The samples were applied over the surface at 10 μL/min for 120 seconds with a regeneration time of 240 seconds. After each injection, the flow delivery system was washed with 50% DMSO to avoid possible binding of molecules. To remove all remaining samples, the surface was regenerated with 10 mM glycine-HCl buffer, pH 2.1. The data obtained was analysed with the Biacore T200 Evaluation Software 2.0 (GE Healthcare). The K_d_ values were obtained from steady state fitting of the response-concentration plot to the equilibrium curves.

### Cell culture

HL-60 cells were maintained in RPMI-1640 culture medium with L-glutamine (Gibco, Life Technologies Corporation, Gaithersburg, Md.), containing 10% foetal bovine serum (Gibco, Life Technologies Corporation), 100 units of penicillin/ml, and 100 μg of streptomycin/ml (Gibco, Life Technologies Corporation) in an incubator with 5% CO_2_ and 95% humidity at 37 °C. Cells in the exponential growth phase were used for experiments.

### MTT assays

HL-60 cells (2 × 10^4^ cells) were plated into 96-well culture plates and treated in triplicate with or without the compounds identified in the CD assays. The compounds were added from stock solutions in DMSO and the final concentration of DMSO in the medium was 0.2%. After 72 h, 20 μL 5 mg/ml MTT (Molecular Probes, Life Technologies Corporation) was added to each well and incubated for 3 h. After the incubation, medium containing drug and MTT was removed, 200 μL DMSO was added to the cells, and the plate was shaken for 5 min. The number of viable cells was assessed by spectrophotometry at 570 nm using a BioTek Synergy4 microplate reader and calculated as the percentage of absorbance of treated cells relative to that of solvent controls. Results were expressed as a percentage of inhibition and the EC_50_ was calculated using the Hill equation.

### Flow cytometric analysis of cell cycle

HL-60 cells (1 × 10^6^ cells) were plated into 6-well culture plates and treated in triplicate with or without the compounds identified in the CD assays. The compounds were added from stock solutions in DMSO and the final concentration of DMSO in the medium was 0.2%. After 24 h, cells were harvested by centrifugation, washed twice with 1 × PBS, fixed in 70% ice-cold ethanol, and kept at 4 °C overnight. Following additional centrifugation, the fixed cells were washed in 1 × PBS and resuspended in 1X PBS containing 50 μg/ml propidium iodide (Life Technologies Corporation) and 50 μg/ml DNase-free RNase A (Life Technologies Corporation). The cell suspension was incubated in the dark for 30 min at 37 °C and analysed using a BD FACSCanto^TM^ cytometer.

### Quantitative real-time PCR

HL-60 cells (4 × 10^4^ cells) were plated into 96-well culture plates and treated in triplicate with or without the compounds identified in the CD assays. The compounds were added from stock solutions in DMSO resulting in a final concentration of 0.2% DMSO in the medium. After 24 h, cells were harvested and real-time quantitative PCR performed using TaqMan^®^ gene expression assay primers (*CCND2*, *CDK4* and *ACTB*) and the TaqMan^®^ gene expression cells-to-CT^™^ kit (Life Technologies Corporation). Negative control reactions lacking cDNA template were included to assess specificity, and showed no appreciable amplification. The fluorescence intensity is related to the initial number of RNA copies, which can be assessed by determining the threshold cycle (C_T_). The fold changes of *CCND2* and *CDK4* expression levels were plotted against the data derived from DMSO-treated samples, and the expression levels of *ACTB* were used as the internal control.

### Chemical cross-linking

Cross-linking reactions were conducted with 1 μM GST-Max (Sino Biological Inc.) or GST as the control with or without 100 μM c-Myc_370–409_ in the presence or absence of different PKUMDL-YC-1205 concentrations in reaction buffer (100 mM sodium phosphate, 150 mM sodium chloride, pH 7.2). c-Myc_370–409_ and PKUMDL-YC-1205 were added from stock solutions in DMSO and the final concentration of DMSO in the buffer was 10%. These samples were treated in duplicate. Freshly prepared 10 mM stock solutions of the homobifunctional amine-reactive N-hydroxysuccinimide ester EGS (Sigma-Aldrich, St Louis, MO) in DMSO were added in 500-fold molar excess to the GST-Max or GST solution. The reactions were conducted at room temperature and quenched after 30 min with 500 mM Tris, pH 7.5 to a final concentration of 50 mM Tris. Samples were boiled with loading buffer and resolved by 10% SDS-PAGE. After electrotransfer of the proteins to a PVDF membrane (Millipore Corporation, Billerica, MA), the membranes were incubated with a 1:200 dilution of anti-Max rabbit polyclonal antibody (Santa Cruz Biotechnology Inc., Santa Cruz, CA) or anti-GST rabbit polyclonal antibody (Santa Cruz Biotechnology Inc.). Next, the blots were probed with horseradish peroxidase-conjugated goat anti-rabbit IgG antibody (Santa Cruz Biotechnology Inc.) and proteins were detected by chemiluminescence (Vigorous Biotechnology, Beijing, China).

### Nuclear Magnetic Resonance (NMR) spectroscopy

All NMR experiments were performed at 25 °C using a Bruker Advanced 500 or 800 MHz spectrometer equipped with Cryo probes. A 0.5 mM c-Myc_370–409_ sample in 5 mM PBS, pH 6.3, 90% H_2_O, 10% D_2_O buffer was prepared for backbone and side-chain ^1^H resonance assignment using ^1^H-^1^H NOESY and TOCSY experiments. Samples containing 0.1 mM c-Myc_370–409_ in the absence and presence of 0.2 mM PKUMDL-YC-1205 were prepared in 5 mM PBS, pH 6.3, 1% DMSO-d_6_, and 99% D_2_O. Samples containing 2 mM PKUMDL-YC-1205 with or without 0.04 mM cMyc_370–409_ were prepared in DMSO-d_6_ for STD NMR experiments.

TOCSY spectra were acquired with a relaxation delay of 1.5 s, 60 ms TOCSY mixing time. NOESY spectra were acquired with a relaxation delay of 2 s, 150 ms NOESY mixing time. All experiments used shaped pulses for water suppression, and the 2D spectra were processed by Bruker^®^ Topspin software to 2048 × 1024 points and analysed by CcpNmr software^53^.

STD NMR experiments were performed with a train of 50 ms Gaussian-shaped saturating pulses at 140 Hz power for 3 s with “on” resonance saturation at 0.84 ppm and “off” resonance saturation at 30 ppm. (The relaxation delay was 2 s before the saturating pulses.) The number of scans was 32768 and the spectral width was 16 ppm. STD NMR Spectrum was recorded using a Bruker Advanced 500 spectrometer equipped with Cryo probes and processed by Bruker^®^ Topspin software.

### Molecular dynamics simulations and analysis

To investigate the interactions between c-Myc_370–409_ and two enantiomers of 10074-A4, PKUMDL-YC-1205 and AJ-292/41944612, molecular dynamics simulations for each complex structure were conducted with explicit water using the Amber molecular dynamics package^54^ and AMBER99SB force field^55^. The initial complex structures were built by molecular docking using the Glide^32^,^33^ software in SP mode. The systems were neutralized by adding ions and immersed in an octahedral periodic box with TIP4P-Ew^56^ water molecules with a closeness parameter of 8 Å away from the boundary of any atoms. The system was minimized using the steepest descent and conjugate gradient minimization approaches. After minimization, all systems were heated from 0 K to 298 K in 50 ps, followed by 50 ps density equilibration at 298 K with weak restraints on the complex. The systems were run with constant pressure and temperature (NPT ensemble mode) with periodic boundary conditions. For each complex, five 100-ns simulations were performed. Amber Tools was used to analyse the simulation trajectories. VMD^57^ was used to analyse the interactions between c-Myc_370–409_ and compounds with a cut off of 5 Å. The conformations from the five simulations of c-Myc_370–409_ with PKUMDL-YC-1205 were clustered into eight clusters using the average-linkage algorithm with RMSD being the similarity metric.

## Additional Information

**How to cite this article**: Yu, C. *et al.* Structure-based Inhibitor Design for the Intrinsically Disordered Protein c-Myc. *Sci. Rep.* **6**, 22298; doi: 10.1038/srep22298 (2016).

## Supplementary Material

Supplementary Information

## Figures and Tables

**Table 1:** Docking scores and activities of compounds that bind the disordered bHLH-LZ domain of c-Myc.

		Docking Score (kcal/mol)			Cell-free Assays		Cell-based Assays		
Compound		Apo1	Apo2	Holo1	CD/ApparentK_d_ (μM)	SPR/K_d_(μM)	Growth Inhibition/EC_50_(μM)	S-Phase Increase	Transcriptional Activity Inhibition
PKUMDL-YC-1101		−6.596*	−5.303	−4.224	94 ± 21	0.28 ± 0.14	>100	No	No
PKUMDL-YC-1201		−5.170	−4.007	−5.504*	70 ± 11	17.2 ± 7.2	105 ± 10	Yes	Yes
PKUMDL-YC-1202		−5.071	−4.474	−5.837*	235 ± 141	112 ± 81	>100	No	No
PKUMDL-YC-1203		−4.343	−4.265	−5.175*	346 ± 245	32 ± 18	6.9 ± 1.1	Yes	Yes
PKUMDL-YC-1204		−4.282	−4.001	−5.244*	90 ± 15	0.55 ± 0.14	8.80 ± 0.26	Yes	Yes
PKUMDL-YC-1205		−4.734	−3.483	−5.360*	61.8 ± 0.7	18 ± 12	40.0 ± 1.9	Yes	Yes
PKUMDL-YC-1301		—			230 ± 88	77 ± 22	>100	Yes	Yes
10074-A4	R	−5.086*	−3.734	−4.347	128 ± 46	36.3 ± 9.0	15.1 ± 2.3	Yes	Yes
	S	−5.253*	−3.966	−4.122					
10058-F4		—			—	—	40.3 ± 2.7	Yes	Yes

^*^Main binding site.
