# Supplementary material for: Structure-based Inhibitor Design for the Intrinsically Disordered Protein c-Myc
Source: Sci Rep. 2016 Mar 2;6:22298. doi: 10.1038/srep22298 (PMC4773988; doi:10.1038/srep22298)
Supplement: Supplementary Information [file srep22298-s1.pdf]

**Structure-based Inhibitor Design for the Intrinsically Disordered Protein c-Myc**

Chen Yu, Xiaogang Niu, Fan Jin, Zhirong Liu, Changwen Jin, and Luhua Lai

**Supporting Information**

1. Supplementary Figures 1-9
2. Table S1 Ranks and library IDs of identified hits in virtual screening

# Supplementary Figures 1-9

**Figure S1 Circular dichroism (CD) spectra of c-Myc$_{370-409}$ with different compounds.**

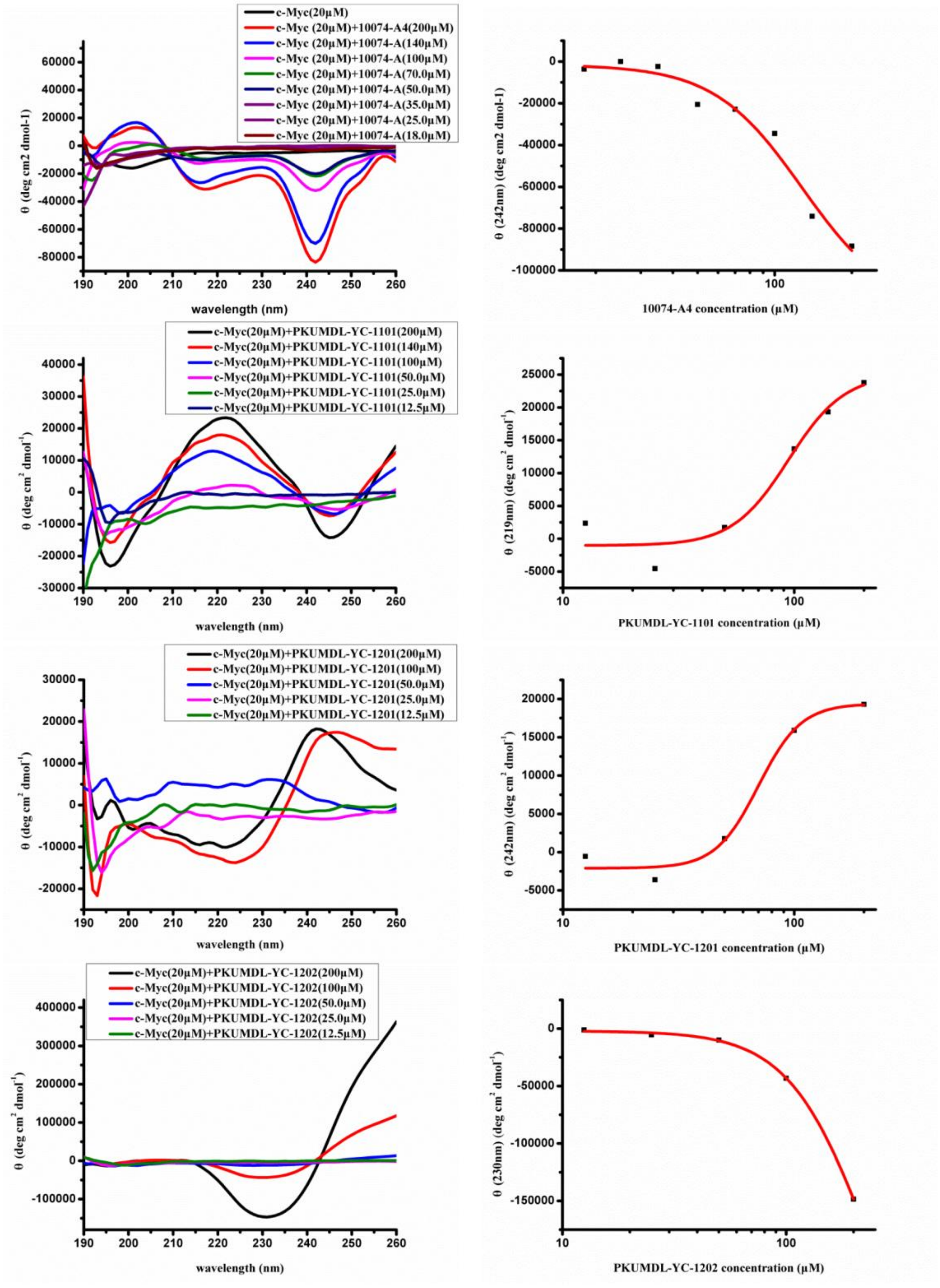

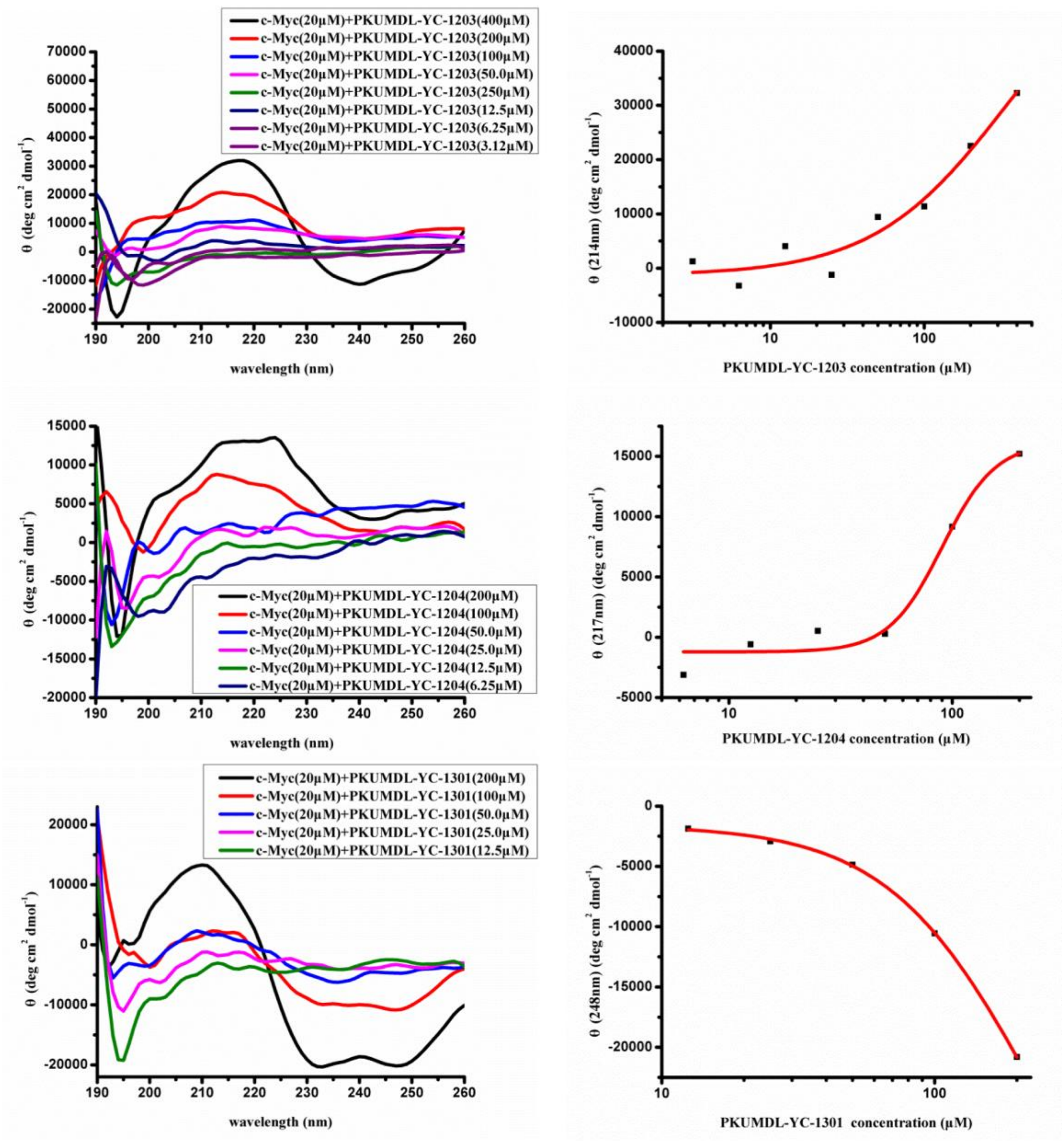
c-Myc(20μM)+PKUMDL-YC-1203(400μM)
c-Myc(20μM)+PKUMDL-YC-1203(200μM)
c-Myc(20μM)+PKUMDL-YC-1203(100μM)
c-Myc(20μM)+PKUMDL-YC-1203(50.0μM)
c-Myc(20μM)+PKUMDL-YC-1203(250μM)
c-Myc(20μM)+PKUMDL-YC-1203(12.5μM)
c-Myc(20μM)+PKUMDL-YC-1203(6.25μM)
c-Myc(20μM)+PKUMDL-YC-1203(3.12μM)
θ (deg cm$^2$ dmol$^{-1}$)
wavelength (nm)
θ (214nm) (deg cm$^2$ dmol$^{-1}$)
PKUMDL-YC-1203 concentration (μM)
c-Myc(20μM)+PKUMDL-YC-1204(200μM)
c-Myc(20μM)+PKUMDL-YC-1204(100μM)
c-Myc(20μM)+PKUMDL-YC-1204(50.0μM)
c-Myc(20μM)+PKUMDL-YC-1204(25.0μM)
c-Myc(20μM)+PKUMDL-YC-1204(12.5μM)
c-Myc(20μM)+PKUMDL-YC-1204(6.25μM)
θ (deg cm$^2$ dmol$^{-1}$)
wavelength (nm)
θ (217nm) (deg cm$^2$ dmol$^{-1}$)
PKUMDL-YC-1204 concentration (μM)
c-Myc(20μM)+PKUMDL-YC-1301(200μM)
c-Myc(20μM)+PKUMDL-YC-1301(100μM)
c-Myc(20μM)+PKUMDL-YC-1301(50.0μM)
c-Myc(20μM)+PKUMDL-YC-1301(25.0μM)
c-Myc(20μM)+PKUMDL-YC-1301(12.5μM)
θ (deg cm$^2$ dmol$^{-1}$)
wavelength (nm)
θ (248nm) (deg cm$^2$ dmol$^{-1}$)
PKUMDL-YC-1301 concentration (μM)

**Figure S2 Surface plasmon resonance (SPR) assay results of different compounds.**

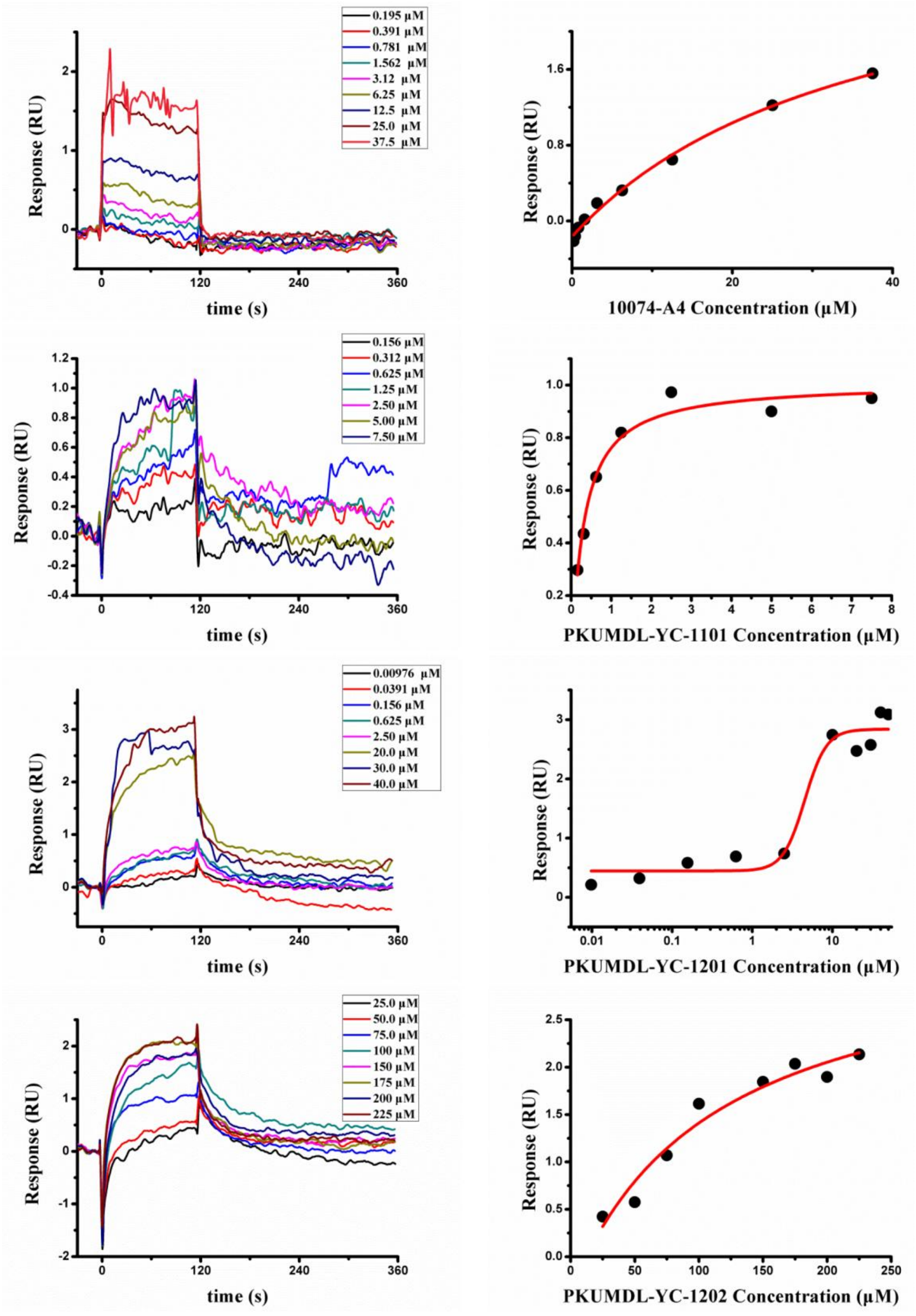

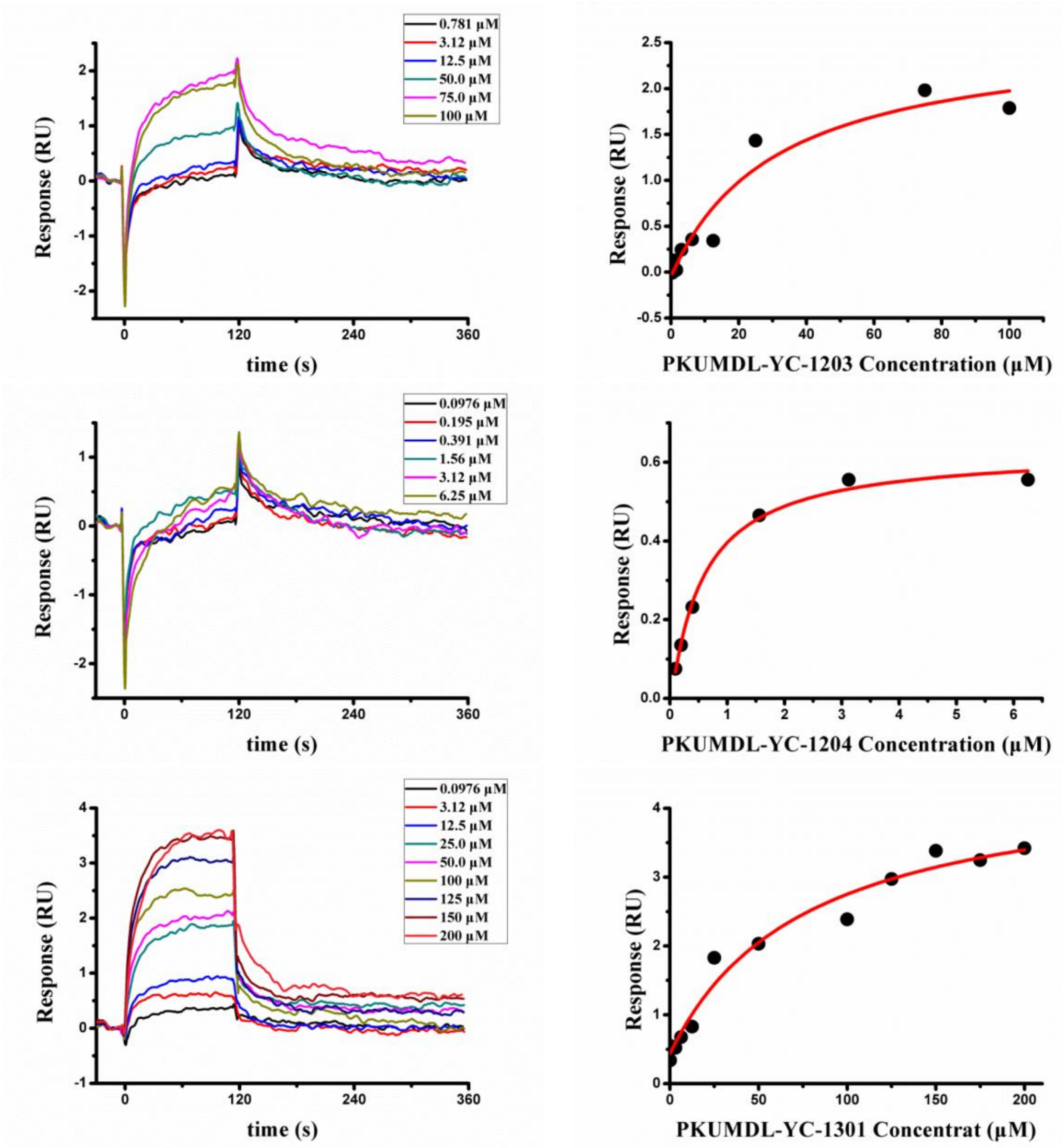
0.781 μM
3.12 μM
12.5 μM
50.0 μM
75.0 μM
100 μM
Response (RU)
time (s)
Response (RU)
PKUMDL-YC-1203 Concentration (μM)
0.0976 μM
0.195 μM
0.391 μM
1.56 μM
3.12 μM
6.25 μM
Response (RU)
time (s)
Response (RU)
PKUMDL-YC-1204 Concentration (μM)
0.0976 μM
3.12 μM
12.5 μM
25.0 μM
50.0 μM
100 μM
125 μM
150 μM
200 μM
Response (RU)
time (s)
Response (RU)
PKUMDL-YC-1301 Concentrat (μM)

**Figure S3 HL-60 cells growth inhibition of different compounds.** Data represent the mean ± standard deviation of three independent experiments.

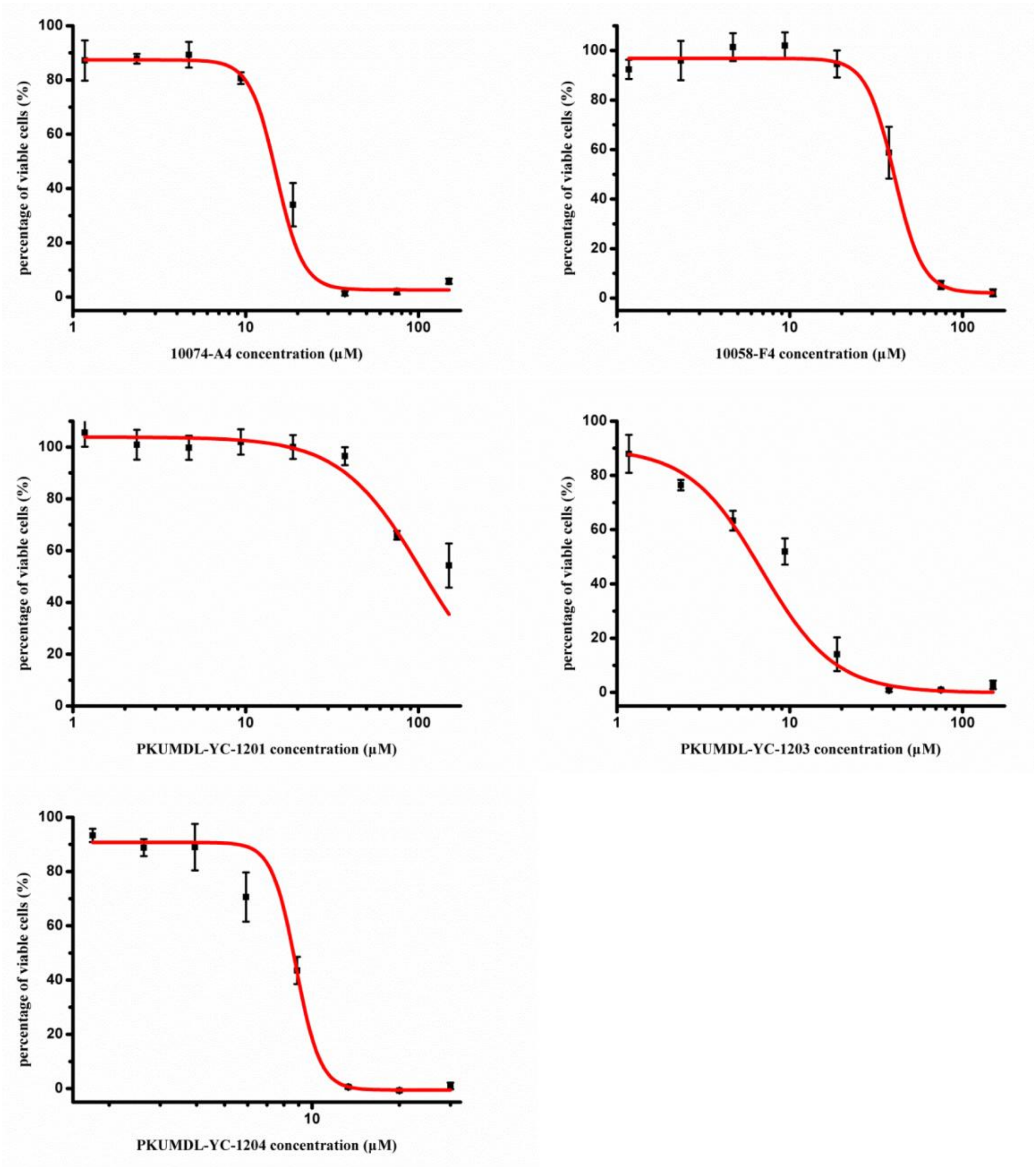

**Figure S4 Compounds changed HL-60 cell cycle.** Data represent the mean ± standard deviation of three independent experiments. *$p < 0.05$, **$p < 0.01$.

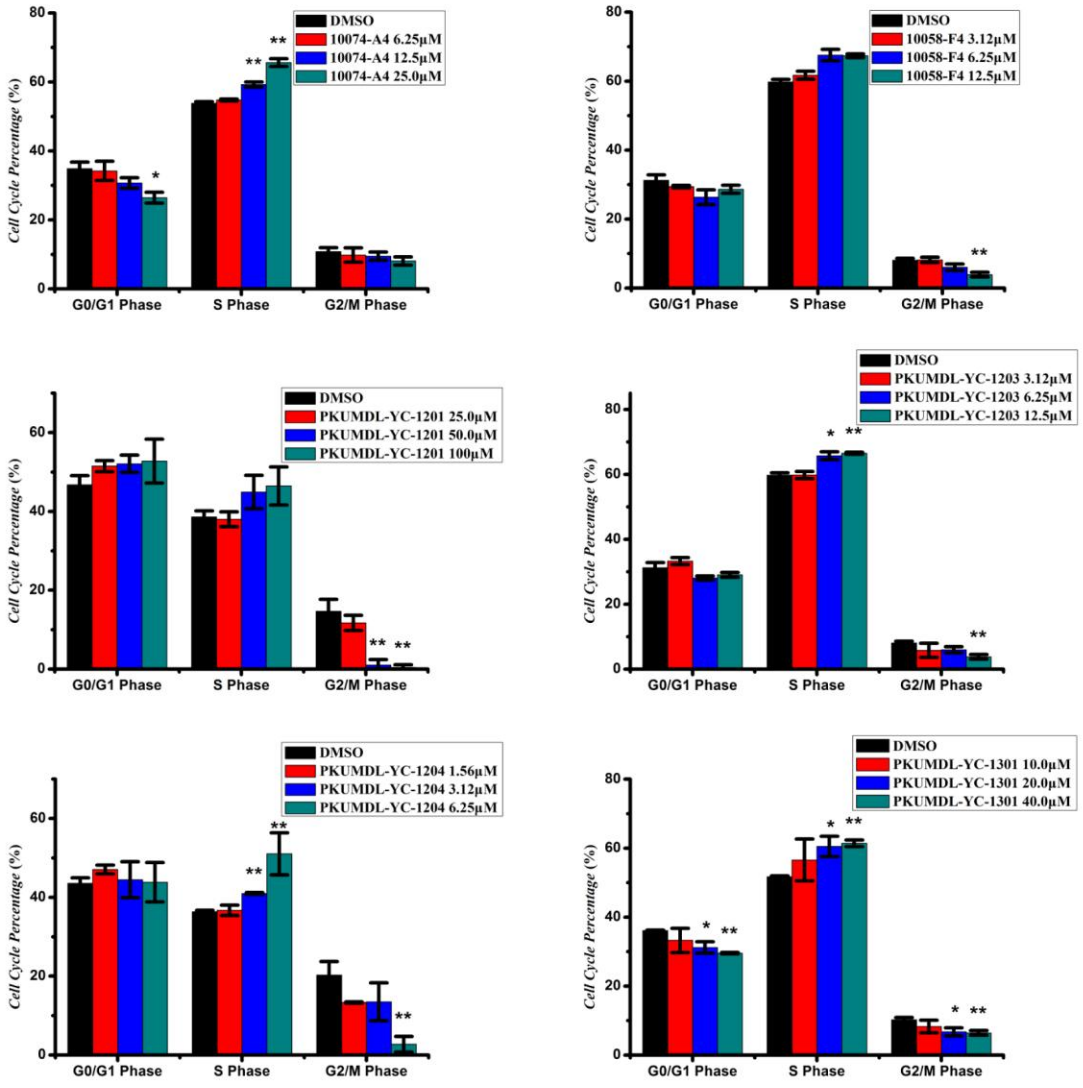

**Figure S5 PKUMDL-YC-1205's non-specific binding to GST-Max.**

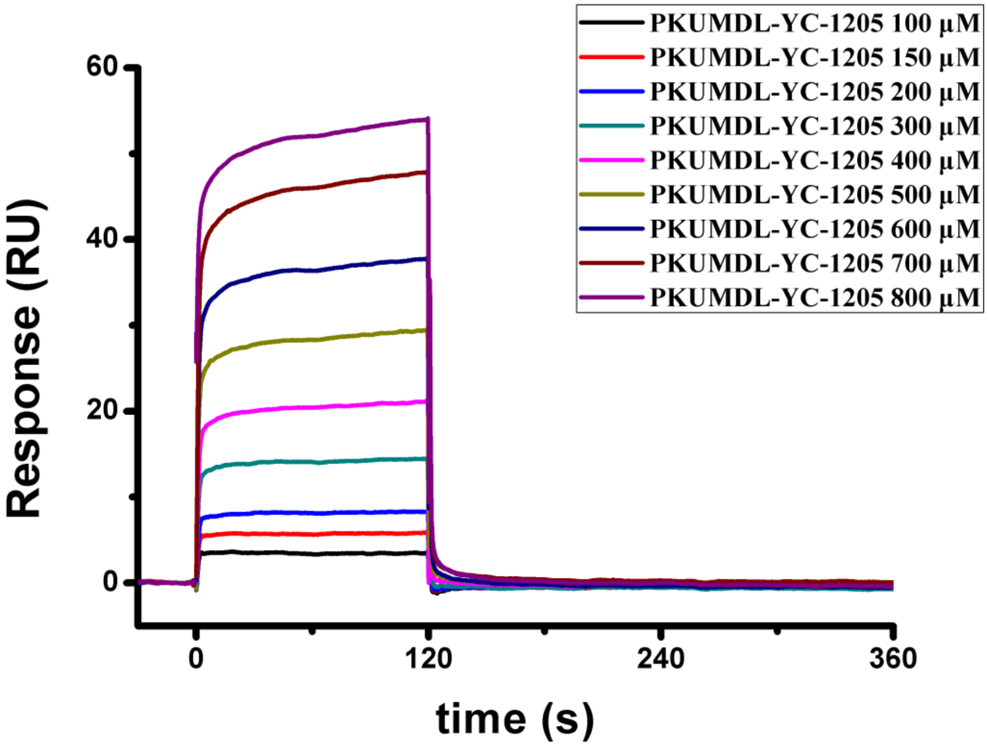


**Figure S6 PKUMDL-YC-1205 had no effect on GST dimerization equilibrium.** (A) Chemical cross-linking and anti-GST western blot results. (B) Blackness integrals of GST homodimer percentage are shown as a histogram. Data represent the mean ± standard deviation of two independent experiments.

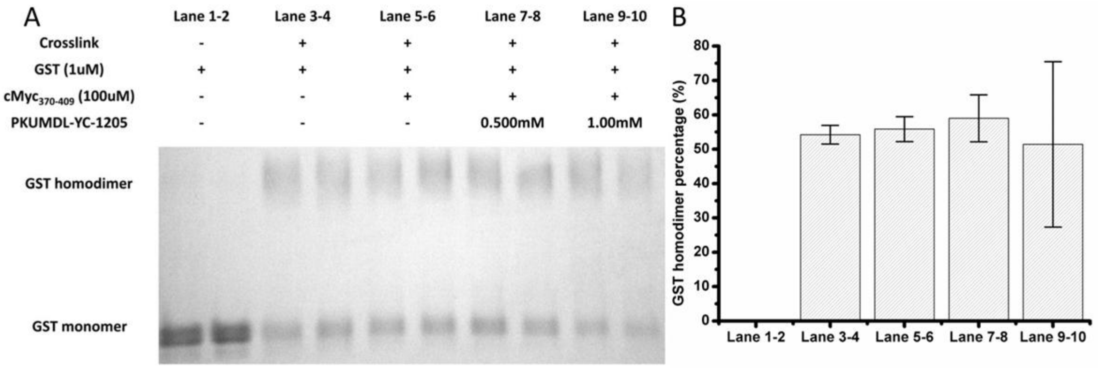

**Figure S7 Overlapped $^{1}$H-$^{1}$H TOCSY spectrum of c-Myc$_{370\text{-}409}$ with (green) and without (red) PKUMDL-YC-1205.**

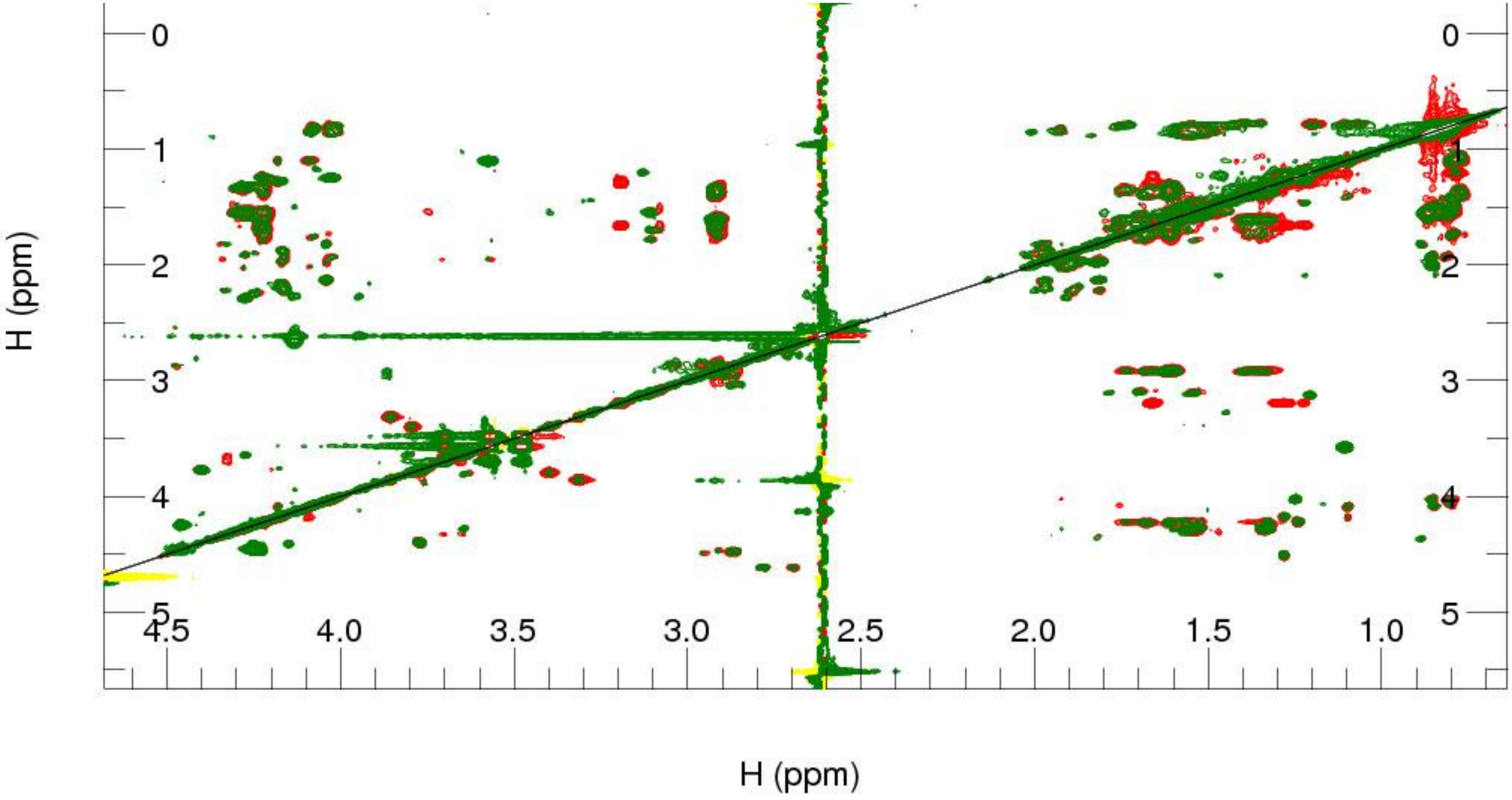

**Figure S8 Interactions between compounds and c-Myc$_{370\text{-}409}$ in molecular dynamic simulations.** Black squares represent the interactions between PKUMDL-YC-1205 (A), 10074-A4 (S form, B), AJ-292/41944612 (C) and the residues of c-Myc$_{370\text{-}409}$. Five independent 100-nanosecond simulations are represented.

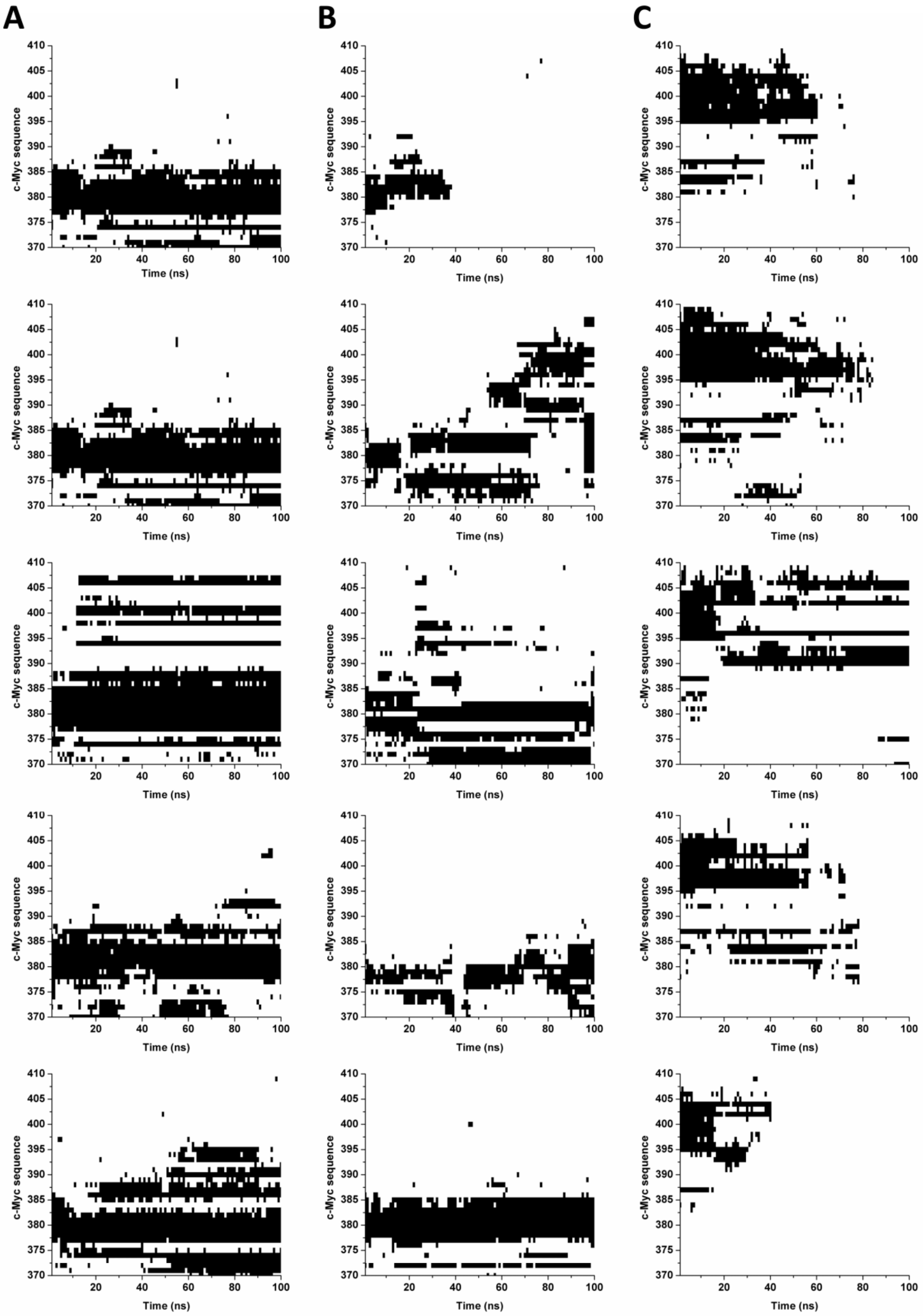
A
B
C
c-Myc sequence
Time (ns)

**Figure S9 Docking Modes of Compounds to c-Myc$_{370-409}$.** PKUMDL-YC-1201 (A), PKUMDL-YC-1202 (B), PKUMDL-YC-1203 (C), PKUMDL-YC-1204 (D) are represented in pink and Holo conformation of the c-Myc$_{370-409}$ is represented in green.

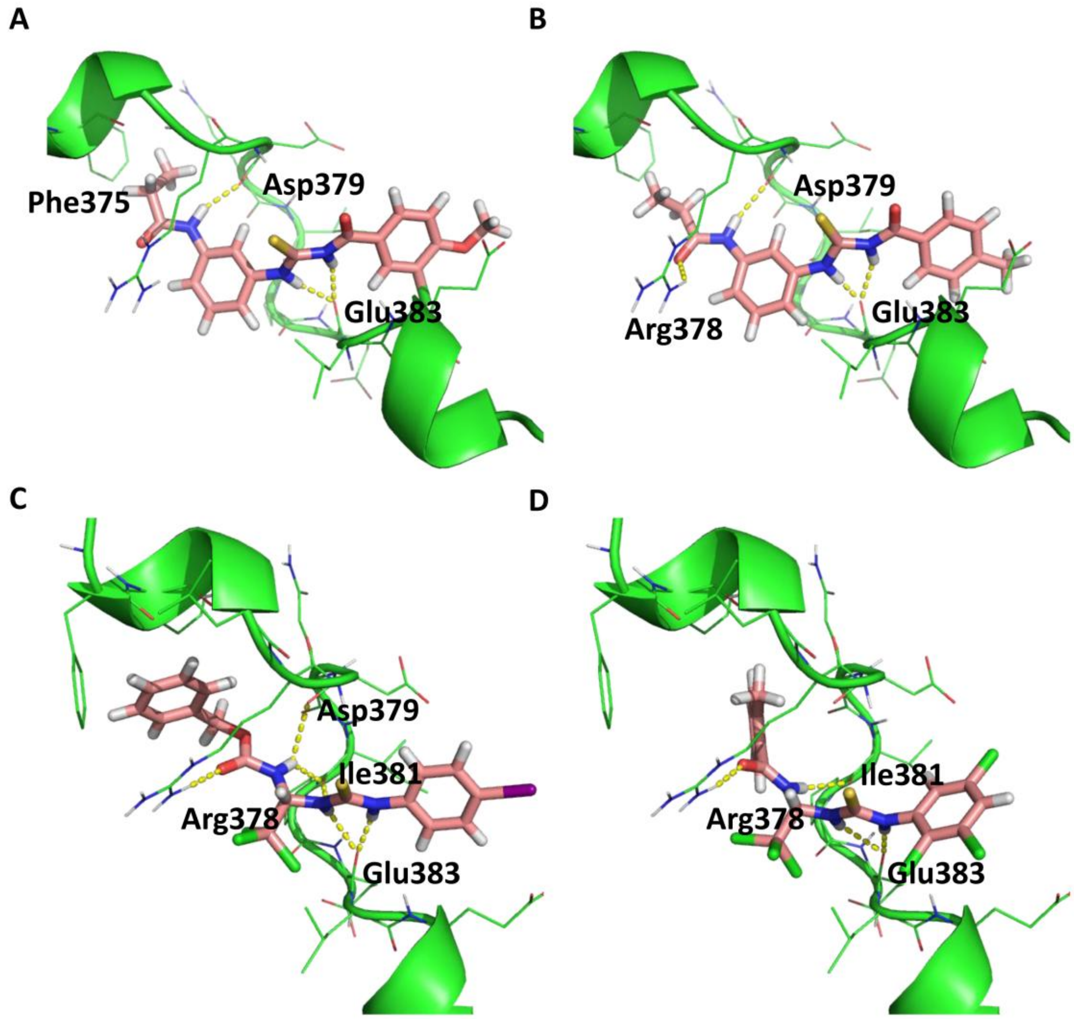

**Table S1 Ranks and library IDs of identified hits in virtual screening**

SPECS libarary: 197,211 compounds

DCSD libarary: 4,728 compounds

Total: 201,939 compounds

| Compound | Cavity | Rank | Library ID |
|---|---|---|---|
| **PKUMDL-YC-1101** | **Apo1** | **6** | **AG-205/36494016** |
| **PKUMDL-YC-1201** | **Holo1** | **19** | **AG-690/15430337** |
| **PKUMDL-YC-1202** | **Holo1** | **5** | **AG-690/15430703** |
| **PKUMDL-YC-1203** | **Holo1** | **63** | **AG-690/33250021** |
| **PKUMDL-YC-1204** | **Holo1** | **51** | **AH-034/07821020** |
| **PKUMDL-YC-1205** | **Holo1** | **32** | **DCSD002857** |
| **PKUMDL-YC-1301** | **Analog of 10074-A4** | **9** | **AH-487/41976595** |
